# Complete Genome Sequence of *Campylobacter jejuni* RM1285, a Rod-Shaped Morphological Variant

**DOI:** 10.1128/genomeA.01361-15

**Published:** 2015-11-25

**Authors:** Nereus W. Gunther, James L. Bono, David S. Needleman

**Affiliations:** a U.S. Department of Agriculture, Agricultural Research Service, Wyndmoor, Pennsylvania, USA; b U.S. Department of Agriculture, Agricultural Research Service, Clay Center, Nebraska, USA

## Abstract

*Campylobacter jejuni* is a spiral shaped Gram-negative food-borne bacterial pathogen of humans found on poultry products. Strain RM1285 is a rod-shaped variant of this species. The genome of RM1285 was determined to be 1,635,803 bp, with a G+C content of 30.5%.

## GENOME ANNOUNCEMENT

*Campylobacter* spp. are responsible for the greatest number of food-borne bacterial gastrointestinal disease cases annually in the developed world (1–3). The incidence of *Campylobacter* infections in the United States has increased by ~14% from data collected in 2006 and 2008 compared to 2014 (4). Poultry products have been historically implicated as a common route for the introduction of *Campylobacter* into the food supply (5–8). Testing in the European Union has suggested that over three-quarters of all broiler carcasses are contaminated with some level of *Campylobacter* (9).

*Campylobacter jejuni* strain RM1285 is an environmental isolate derived from the exudate of a commercial chicken breast acquired from a retail source that was supplied to our laboratory by Robert Mandrell (ARS, Albany, CA). It was observed that morphologically, RM1285 is rod shaped compared to the spiral form common to most other *C. jejuni* strains. Additionally, strain RM1285 demonstrated increased resistance to inactivation by high hydrostatic pressure compared to other *C. jejuni* strains.

The RM1285 genome was sequenced with a PacBio RS II system (Pacific Biosciences, Menlo Park, CA). In this process, the SMRT Analysis software suite was utilized for subread filtering and the Celera Assembler version 8.1 for assembly and error correction (10, 11). Quiver was used to polish the resulting contig, and Geneious version 7.1.5 (Biomatters, Auckland, New Zealand) was used to trim the overlapping ends and reorient the contig, after which Quiver was applied again to verify the trimming and reorienting of the contig (10). The assembly resulted in consensus accuracy of 99.9999%, with a coverage of 20×. Once fully closed, the genome of RM1285 consisted of a single chromosome of 1,635,803 bp in size without any observed extrachromosomal elements.

Of particular interest in the resulting genome sequence of RM1285 was the gene *pgp1*, which codes for a peptidoglycan dl-carboxypeptidase. When the *pgp1* gene sequence is compared to previously sequenced *pgp1* genes from other *C. jejuni* strains, it is observed that a single adenine deletion had occurred at the 1,187 nucleotide of the gene. This resulted in a 62-amino acid truncation of the Pgp1 protein of strain RM1285, leading to a nonfunctional peptidoglycan dl-carboxypeptidase. Previous research has demonstrated that a functional *pgp1* gene in conjunction with the *pgp2* gene is essential for the spiral shape of *Campylobacter*, and the inactivation of either gene results in a rod-shaped variant (12, 13). It is not clear if this defect in the *pgp1* gene is also responsible for the increased resistance of the strain to pressure or if it is simply the resulting shape of the variant strain. Although there are several complete *C. jejuni* genomes that have been sequenced and deposited in GenBank, this is the first example of a complete genome sequencing of a rod-shaped variant of *C. jejuni*.

### Nucleotide sequence accession number.

The genome sequence of *C. jejuni* strain RM1285 has been deposited in GenBank under accession no. CP012696.
